# Look After Yourself: Students Consistently Showing High Resilience Engaged in More Self-Care and Proved More Resilient During the COVID-19 Pandemic

**DOI:** 10.3389/fpsyt.2021.784381

**Published:** 2021-12-21

**Authors:** Laura E. Meine, Eike Strömer, Sandra Schönfelder, Eliza I. Eckhardt, Anna K. Bergmann, Michèle Wessa

**Affiliations:** ^1^Department of Clinical Psychology and Neuropsychology, Institute of Psychology, Johannes Gutenberg-University Mainz, Mainz, Germany; ^2^Department of Psychology, University of Zurich, Zurich, Switzerland; ^3^Department of Psychiatry, Psychotherapy and Psychosomatics, Psychiatric University Hospital Zurich, Zurich, Switzerland; ^4^Leibniz Institute for Resilience Research, Research Group Wessa, Mainz, Germany

**Keywords:** self-care, latent class growth analysis, resilience, COVID-19 pandemic, mental health

## Abstract

The COVID-19 pandemic has prompted severe restrictions on everyday life to curb the spread of infections. For example, teaching at universities has been switched to an online format, reducing students' opportunities for exchange, and social interaction. Consequently, their self-reported mental health has significantly decreased and there is a pressing need to elucidate the underlying mechanisms—ideally considering not only data collected during the pandemic, but also before. One hundred seventeen German university students aged 18-27 were assessed for known resilience factors (optimism, self-care, social support, generalized self-efficacy) and subsequently completed surveys on stress experiences and mental health every 3 months over a period of 9 months before the outbreak of the pandemic and once during the first lockdown in Germany. For each timepoint before the pandemic, we regressed participants' mental health against the reported stressor load, such that the resulting residuals denote better or worse than expected outcomes, i.e., the degree of resilient functioning. We then tested whether different expressions in the resilience factors were predictive of distinct resilient functioning trajectories, which were identified through latent class growth analysis. Finally, we investigated whether trajectory class, resilience factors, and perceived stress predicted resilience during the pandemic. Results show rather stable resilient functioning trajectories, with classes differing mainly according to degree rather than change over time. More self-care was associated with a higher resilient functioning trajectory, which in turn was linked with the most favorable pandemic response (i.e., lower perceived stress and more self-care). Although findings should be interpreted with caution given the rather small sample size, they represent a rare examination of established resilience factors in relation to resilience over an extended period and highlight the relevance of self-care in coping with real-life stressors such as the pandemic.

## Introduction

On March 11, 2020, in response to the rapidly increasing number of cases and growing list of affected countries, the World Health Organization (WHO) declared the coronavirus disease 2019 (COVID-19) a global pandemic (1). Governments worldwide began imposing restrictions on everyday social life to curb the spread of infections. Germany entered its first lockdown in mid-March 2020, closing all non-essential stores, cultural and sports facilities, restaurants, bars, kindergartens, schools, universities, and banning public meetings of more than two people (2, 3). For most, these measures meant an abrupt and serious change in their habits and lifestyle. Although restrictions were gradually lifted in the beginning of May 2020 (4), many measures remained in place or were reintroduced over the course of at least 1.5 years as the country navigated further waves of the pandemic (5). Because of its pervasive impact, the pandemic has been described as a complex, multidimensional stressor that disrupts individuals' daily lives as well as social systems in general, prevents access to protective factors, and has no foreseeable end (6). In line with this, many studies have shown increases in mental health problems and worsening of pre-existing conditions (7, 8). Vulnerable populations include university students whose elevated and rising prevalence rates of depression and anxiety have previously been recognized as a growing problem (9–11). Indeed, evidence from cross-sectional studies investigating students during the pandemic in, e.g., China (12), Spain (13), Germany, and Egypt (14), as well as Italy (15) showed alarming rates of mental health problems, psychological distress, depressive symptoms, and significantly higher levels of psychopathology compared to general workers, respectively. Matos Fialho et al. (16) surveyed over 5,000 German students and reported a perceived increase in workload which was associated with significant stress and worry. However, results from longitudinal studies that include assessments prior to the pandemic (and therefore can investigate changes within the same individuals) are less clear. While some report a pandemic-related rise in mental health problems among students [e.g., (17, 18)], others did not observe a meaningful increase (19, 20). Previous research on mental health symptom trajectories following adversity has shown that resilience, or the maintenance of mental health, is, in fact, the most common response (21). Researchers in the field of resilience have advocated for more investigations of protective features and predictors of good mental health in the face of significant pandemic-related stress (22, 23). Factors that have been established as resilience-promoting include optimism (24), social support (25, 26), perceived self-efficacy (27, 28), and self-care (29, 30).

So far, studies examining the impact of the COVID-19 pandemic on students' mental health focused primarily on identifying risk factors, and these efforts are often hampered by the lack of assessments prior to the outbreak of the pandemic. Here, we analyzed longitudinal data collected both during and before the first pandemic-related lockdown in Germany. Specifically, we aimed at predicting students' resilient functioning during the pandemic as a function of previous resilience trajectories, aforementioned resilience-promoting factors, and perceived stress. In addition, we investigated trajectory class-dependent differences in resilience factors at baseline. We expected distinct differences in students' resilient functioning trajectories over the multiple pre-pandemic assessments (e.g., decreasing, increasing, or stable trajectories). Based on the literature, we hypothesized that differences in optimism, social support, perceived self-efficacy, and self-care would distinguish putative resilience trajectory types. With respect to the pandemic, we assumed that it was associated with increased stress and poorer mental health among students. We expected that a more favorable resilient functioning trajectory (i.e., consistently high or increasing levels), higher expression in the resilience factors, and lower perceived stress would be predictive of better resilience during the lockdown.

## Materials and Methods

### Participants

We used data from a large-scale longitudinal intervention study in which university students were assigned to either a resilience training or a wait-list control group. Here, we considered only the 316 control participants who did not undergo any training. These participants came from two different cohorts and were matched according to data collection time points (see section Procedure for details). Since we operationalized resilient functioning as the residual resulting from the regression of mental health on experienced stress (see section Data Preparation and Statistical Analyses for details), we only included participants who provided complete data for predictor and criterion at all time points. Thus, the residuals always represent deviations from the expected relationship based on the same population. In addition, three participants had to be excluded from the analysis: one had duplicate data from both cohorts, one had not reported any stressful events at baseline, and one reported an extremely high frequency of microstressors at one time point (> 5 *SD* from sample mean). The final sample comprised 133 students aged 18-27 (75% female, age: *M* = 20.56, *SD* = 1.76; 67% belonging to the later cohort), all of whom were fluent in German, had not received psychotherapeutic or psychiatric treatment within the last 5 years, reported no regular alcohol or drug use, no self-harming behavior or suicidal ideation within the last 6 months, and had not experienced a major traumatic event. Nearly all these participants completed a follow-up online survey, yielding a sample of 117 students (74% female, age: *M* = 21.69, *SD* = 1.76; 66% belonging to the later cohort) for analyses focusing on resilient functioning during the pandemic. Only one participant reported having tested positive for COVID-19 and experiencing symptoms including fever which were treated at home. 6% stated they belonged to a risk group for a severe course of the disease and 5% had been quarantining at home. This study was approved by the ethics committee of the Institute of Psychology, Johannes Gutenberg-University, Mainz, Germany (2018-JGU-psychEK-001, 27/03/2018), and was conducted in accordance with the Declaration of Helsinki.

### Procedure

**Figure 1:**
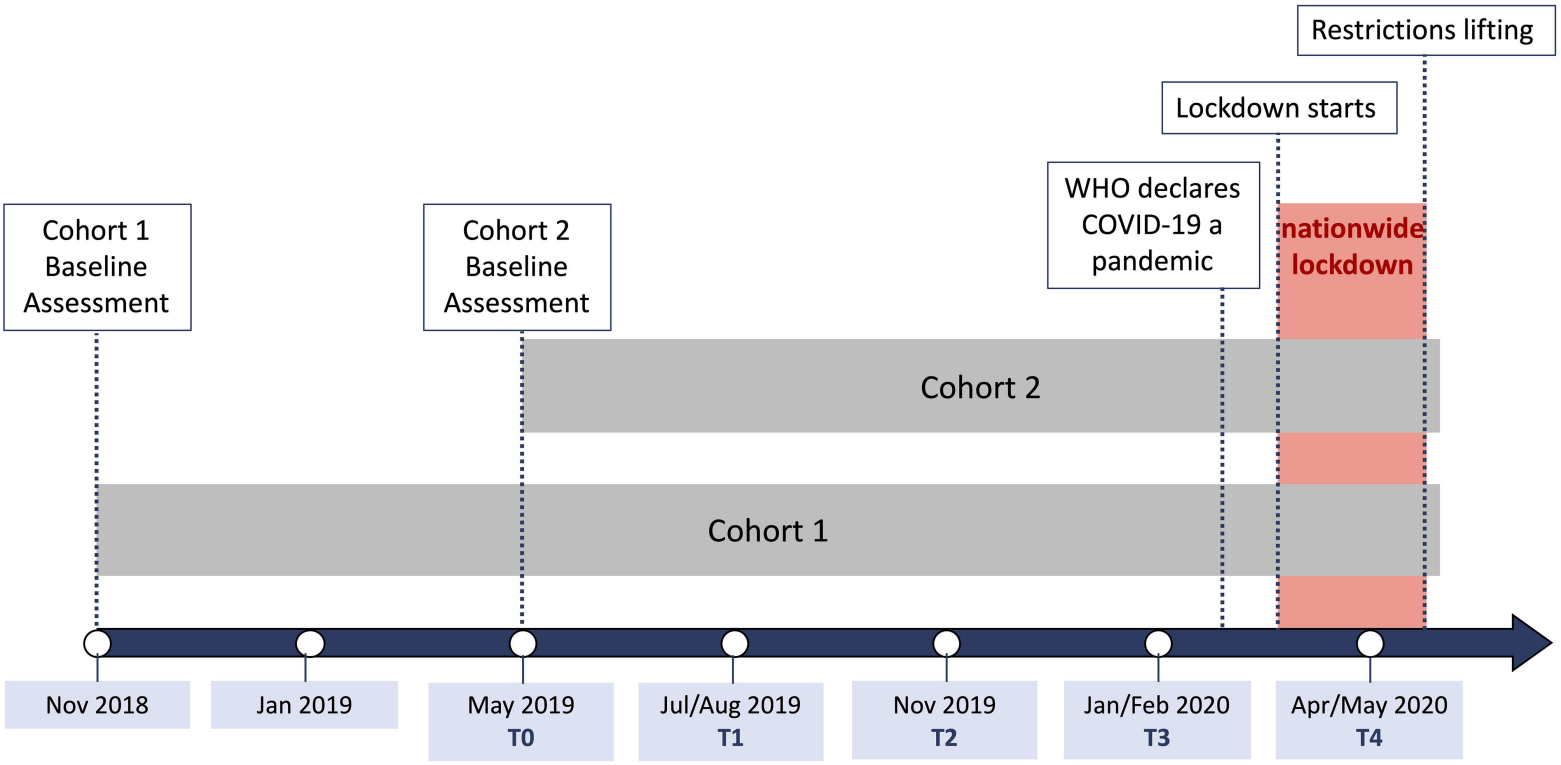
Timing of data collection.

Participants took part in an initial 1.5 h on-site assessment (T0) in the Mainz Behavioral and Experimental Laboratory (MABELLA) at Johannes Gutenberg-University Mainz. Upon arrival, they received information on the study and planned procedures and gave written informed consent. Participants then provided self-report data on demographics and established resilience factors; namely, optimism, self-care, social support, and generalized self-efficacy. They also completed questionnaires measuring stressor load, mental health, and well-being. Following this baseline assessment, participants were asked to fill out the latter questionnaires online every 3 months over a 9-month period before the outbreak of the pandemic (T1-T3). During the first pandemic-related lockdown in Germany, we conducted another online follow-up (T4; 27 April to 13 May 2020) in which participants were again asked to report on their stress experiences and mental health, but this time against the specific backdrop of the ongoing pandemic. In addition, we re-assessed the aforementioned resilience factors. Upon completion of each session, participants were remunerated with 15 €. Note that, in order to match the data of the two cohorts by time of assessment and to ensure an equal number of assessments before the pandemic, we had to disregard the first two time points of the first cohort. We accounted for possible effects of previous sessions in this cohort by including cohort as a covariate in our analyses. Figure 1 illustrates the timing of data collection for both cohorts separately and relates it to pandemic events. The following section provides details on the questionnaires we used for this study.

### Questionnaires

First, we describe the questionnaires used for the resilience factors listed before at T0. Optimism was measured using the corresponding three-item subscale of the German version of the revised Life Orientation Test [LOT-R; (31); original English version by (32)], which has an acceptable internal consistency (Cronbach's α = 0.69). Self-care was assessed using the mean across all 12 items of the Hamburg Self-Care Questionnaire [HamSCQ; (33)], subsuming the subscales pacing (i.e., mindful handling of oneself and one's limits) and positive experience (i.e., accepting and enjoying positive behaviors; Cronbach's α > 0.9 for both scales). The 14-item short form of the Social Support Questionnaire [F-SozU-K14; (34); Cronbach's α = 0.94] was included as a measure of social support, and the German version of the Generalized Self-Efficacy Scale [GSE; (35); 10 items; Cronbach's α > 0.7] provided an indicator of generalized self-efficacy.

Second, we list the stress and mental health questionnaires that participants completed at T0-T4. The German version of the Brief Symptom Inventory-18 [BSI-18; (36); original English version by (37)] uses six items each to capture psychological distress in the past week via the subscales somatization, depression, and anxiety. However, here we used the Global Severity Index (GSI) which covers all items and has excellent internal consistency [Cronbach's α = 0.93; (38)]. The German version of the WHO Well-Being Index [WHO-5; (39); original English publication by (40)] was used as another indicator of mental health, comprising five items that refer to the past 2 weeks (Cronbach's α = 0.92). Participants also completed the Mainz Inventory of Microstressors [MIMIS; (41)] which measures the frequency and intensity of 58 microstressors (e.g., commute to work, problem with a pet, time pressure) within the past week. A 27-item life events checklist [(42); adapted from (43)] provided a count of more severe stressors (e.g., death of a friend, law violations, serious financial problems) encountered in the past 3 months. Since the checklist also includes items that may not be perceived as stressful by all participants (e.g., marriage plans, child starting school), we only counted life events if they were rated as at least a bit burdensome (i.e., 1 on a scale ranging from 0 = not at all burdensome to 4 = very burdensome). At T0, participants were instructed to rate all events they had experienced up to that date.

Third, we elaborate on additional questionnaires assessed at T4 (i.e., during the lockdown). These included items on COVID-19 risk group status, infection, symptom severity, and quarantine, as well as a 29-item list of stressors specific to the context of the pandemic (44). For stressors that had occurred to them, participants provided intensity ratings on a scale from 1 (not at all burdensome) to 5 (extremely burdensome) and we calculated stressor count as well as mean scores reflecting stressor burden. We also assessed participants' agreement with government-mandated restrictions and the degree to which they were following official recommendations. For both items, we used a Likert scale ranging from 1 (not at all) to 5 (very). In addition, we measured perceived stress using the German version of the Perceived Stress Scale [PSS; (45); original English version by (46)]. The PSS can be split into the subscales helplessness and self-efficacy, comprising six and four items, respectively. However, here we used the total score across all items as an indicator of general subjective stress level in the past seven days (Cronbach's α = 0.88). Whereas, optimism, self-care, and generalized self-efficacy were re-assessed using the same instruments as at T0, we used the 4-item subscale perceived emotional support of the Berlin Social Support Scales [BSSS; (47); Cronbach's α = 0.81] to assess social support during the pandemic. The BSSS items were presented in the past tense and participants were instructed to refer to the past 4 weeks.

### Data Preparation and Statistical Analyses

We calculated a measure of resilient functioning for each time point T0-T4 based on the following variables: the GSI of the BSI-18, the WHO-5, the frequency of microstressor encounters, and the count of stressful life events. In computing the score, we followed established procedures described in previous publications [e.g., (48–51)]. To obtain a single indicator of both mental health and stress, we first conducted a principal component analysis of the standardized GSI and WHO-5 scores and the standardized microstressor and life events scores, respectively. To match the WHO-5 response format, we used the inverted score of the GSI such that higher values indicated fewer symptoms i.e., a more positive outcome. The extracted first component taken to reflect mental health was then regressed on the first component representing stressor load. The resulting residuals therefore denoted better or worse than expected mental health based on the given stress experience. Hence, we obtained a continuous measure of resilient functioning.

For our investigation of distinct classes of resilient functioning trajectories, we followed instructions by Wickrama et al. (52). Prior to conducting a latent class growth analysis (LCGA) to identify trajectory classes, we determined its appropriateness through univariate growth curve modeling. We fitted an unconditional single growth curve (linear and quadratic) to the resilient functioning scores from T0-T3 and verified adequate model fit (53) based on the root mean square error of approximation (RMSEA ≤ 0.05), the Comparative Fit Index (CFI ≥ 0.95), the Tucker-Lewis Index (TLI ≥ 0.95), and the standardized root mean square residual (SRMR ≤ 0.07). Next, we compared LCGA results for unconditional models with one to five classes, fixing all within-class variances to zero. Unlike LCGA, growth mixture modeling (GMM) does not assume homogeneous growth curves within classes and freely estimates within-class variances. It is therefore generally preferred, but our attempts at such a model failed to converge, perhaps reflecting sample size constraints. We decided to use LCGA to ensure model convergence. We specified 500 random sets of starting values and 10 final optimizations to avoid local maxima (54). The optimal number of trajectory classes was determined by comparing standard fit indices listed below, examining latent class membership probabilities, and considering theoretical interpretability. Lower values of the Akaike information criterion (AIC), the Bayesian information criterion (BIC), and the sample size adjusted BIC (SSABIC) suggest better model fit. Entropy values approaching 1 indicate high classification accuracy, and a significant adjusted Lo-Mendell-Rubin likelihood ratio test (adj. LMR-LRT) and bootstrapped LRT (BLRT) show that adding a class significantly improves model fit (55).

To investigate the different resilience factors assessed at T0 as predictors of latent trajectory class, we performed a multinomial logistic regression analysis. First, we checked for extreme values [above the third quartile plus three times the interquartile range (IQR) or below the first quartile minus three times the IQR; (56)] and for multicollinearity (i.e., correlation coefficients of *r* > 0.70) among predictors. Then we set up our model including gender, age, and cohort as predictors alongside the resilience factors.

We analyzed the impact of the pandemic by conducting paired samples *t*-tests to compare participants' stressor load and mental health before the lockdown (T3) with assessments during the lockdown (T4).

Finally, we used multiple regression to examine the predictive value of trajectory class, resilience factors (re-assessed during the pandemic), and perceived stress on participants' resilient functioning during the lockdown. Class-dependent differences in resilient functioning during the pandemic were further investigated by comparing all groups. We applied Holm-Bonferroni correction for multiple comparisons.

Analyses were mainly performed in R, version 4.0.5 (https://www.r-project.org), latent trajectory classes, however, were identified using Mplus, version 7.3 (57).

## Results

### Resilient Functioning Scores

The principal component analysis conducted for each of the five time points (T0-T4) resulted in components for mental health and stressor load, each of which explained above 60% of variance. As intended, higher values in mental health components indicated better mental health and higher values in stressor load components reflected higher stressor load. Results of the linear regressions performed for each time point showed that stressor load was a significant predictor of mental health (see Supplementary Table 1 for detailed results). Participants reported lower mental health with increasing stressor load. The resulting residuals were taken to reflect participants' level of resilient functioning (see Supplementary Figure 1 for visualization).

### Growth Curve Modeling

A linear growth curve model of the resilient functioning scores from T0-T3 showed excellent fit to the data (CFI = 1.000; TLI = 1.000; RMSEA = 0.000; SRMS = 0.021). Although the mean slope was not significantly different from zero (*p* > 0.05), an intercept-only model (assuming no change in resilient functioning over time) demonstrated much worse data fit (CFI = 0.833; TLI = 0.875; RMSEA = 0.153; SRMS = 0.093). Significant variance of intercept and slope (both *p* < 0.001) also indicated interindividual differences in initial levels of resilient functioning as well as in change over time, suggesting the appropriateness of investigating potentially underlying distinct trajectory classes with LCGA. To test for curvilinear patterns of change, we incorporated a quadratic term into the model, but resulting fit indices showed only slight improvement (CFI = 1.000; TLI = 1.000; RMSEA = 0.000; SRMS = 0.009) and information criteria were higher, suggesting worse model fit (e.g., BIC_linear_ = 1543.654; BIC_quadratic_ = 1557.599). The nested χ^2^ difference test was also not significant ($\chi_{\text{DIFF}}^{2}$ = 0.726 well below critical cut-off value of 7.81, based on α = 0.05 and *df* = 3), therefore we retained the more parsimonious linear model.

### Latent Class Growth Analysis

We compared fit indices of unconditional models with one to five latent classes (Table 1). Decreases in AIC, BIC, and SSABIC across consecutive models, reflected better model fit with increasing number of classes. However, information criteria increased from the four-class to the five-class solution, indicating worse fit of the latter model. Entropy was highest for the three-class solution, although classification accuracy was similar for the four-class solution. In fact, adj. LMR-LRT (*p* = 0.011) and BLRT (*p* < 0.001) indicated significant improvement in model fit for the four-class solution compared to the three-class solution. Moreover, one of the three latent classes contained only eight participants, barely more than the recommended minimum of 5% of the total sample (52). We therefore selected the four-class model which also had high average latent class probabilities (0.93, 0.89, 0.87, and 0.87, for classes 1-4, respectively), meaning that participants were assigned to the latent class to which they were most likely to belong.

**Table 1:** Goodness of fit statistics for one- to five-class models of resilient functioning trajectories.

**Model**	**AIC**	**BIC**	**SSABIC**	**Entropy**	**Adj. LMR-LRT (*p*)**	**BLRT (*p*)**
One-class	1666.932	1684.274	1665.295	–	–	–
Two-class	1556.772	1582.785	1554.317	0.781	<0.001	<0.001
Three-class	1536.484	1571.168	1533.211	0.809	0.431	<0.001
**Four-class**	**1503.506**	**1546.861**	**1499.414**	**0.790**	**0.011**	**<0.001**
Five-class	1504.952	1556.979	1500.042	0.690	0.499	0.460

*AIC, Akaike information criterion; BIC, Bayesian information criterion; SSABIC, sample-size adjusted BIC; Adj. LMR-LRT, adjusted Lo-Mendell-Rubin likelihood ratio test; BLRT, bootstrapped likelihood ratio test. Information for the model ultimately selected were bolded*.

**Figure 2:**
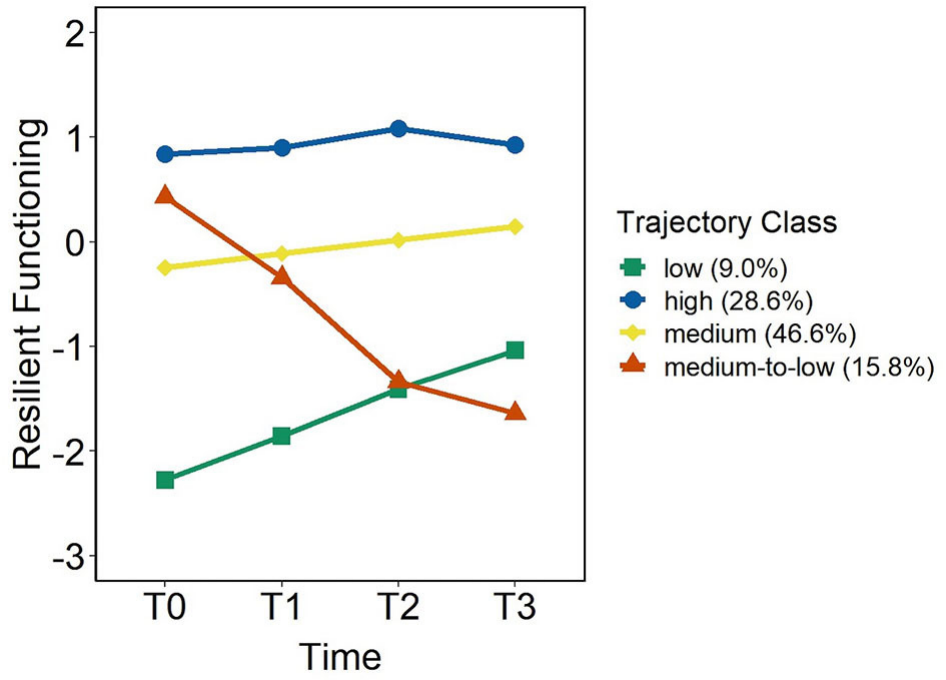
Trajectories of resilient functioning from T0-T3 according to the four-class model.

In the four-class model (Figure 2), the largest class comprised 46.6% of the sample, with participants showing the expected level of resilient functioning (intercept *M* = −0.24 ± 0.11, *p* = 0.032) with a marginal increase over time (slope *M* = 0.13 ± 0.07, *p* = 0.055). Because resilient functioning was operationalized as the residual from the regression of mental health on stressor load, values around zero denote the expected level of mental health given reported stress experience. Therefore, these participants exhibited neither high nor low, but rather expected or “medium” levels. The second-largest class (28.6%) followed a stable trajectory at “high” levels of resilient functioning (intercept *M* = 0.88 ± 0.29, *p* < 0.002; slope *M* = 0.04 ± 0.10, *p* = 0.706), i.e., participants consistently reported better than expected mental health given their stressor load. A third class, “medium-to-low” (15.8%), was characterized by expected levels of resilient functioning at baseline (intercept *M* = 0.34 ± 0.30, *p* = 0.254) and a marked decline over time (slope *M* = −0.70 ± 0.13, *p* <0.001). The last class (9%) included participants at rather “low” levels of resilient functioning (intercept *M* = −2.27 ± 0.41, *p* < 0.001), but whose trajectories indicated some improvement over time (slope *M* = 0.42 ± 0.20, *p* = 0.035).

To better compare the four trajectories, Table 2 provides details on demographics, resilience factors at baseline, and average mental health and stressor load across T0-T3 of each class. In addition, for each class, we plotted the trajectories of the variables from which the resilient functioning score was derived (Supplementary Figure 2).

**Table 2:** Demographics, resilience factors at baseline, average stress and mental health across T0-T3, and resilience factors and perceived stress during the pandemic by class.

	**Trajectory classes**				**Test statistic**	***p***
	**High**	**Medium**	**Medium-to-low**	**Low**		
**Demographics**						
*n* (%)	38 (28.6)	62 (46.6)	21 (15.8)	12 (9.0)		
Cohort (% later cohort)	68.4	56.5	90.5	75.0	$\chi_{\left(3\right)}^{2}$ = 8.73	0.033
Gender (% female)	65.8	77.4	76.2	91.7	$\chi_{\left(3\right)}^{2}$ = 3.72	0.293
Age in years, *M* (*SD*)	20.55 (1.74)	20.35 (1.56)	21.29 (2.26)	20.42 (1.68)	$\chi_{\left(3\right)}^{2}$ = 3.99	0.262
**Resilience factors at baseline**						
Optimism, *M* (*SD*)	9.26 (1.73)	7.87 (2.36)	7.86 (2.54)	6.08 (2.71)	*F*_(129)_ = 6.80	**<0.001**
Self-care, *M* (*SD*)	4.38 (0.42)	3.93 (0.49)	4.19 (0.50)	3.25 (0.94)	$\chi_{\left(3\right)}^{2}$ = 26.16	**<0.001**
Social Support, *M* (*SD*)	4.51 (0.36)	4.35 (0.49)	4.21 (0.58)	3.88 (0.72)	$\chi_{\left(3\right)}^{2}$ = 10.60	0.014
Generalized self-efficacy, *M* (*SD*)	30.45 (3.45)	28.76 (3.27)	27.57 (4.58)	24.83 (3.19)	*F*_(129)_ = 8.54	**<0.001**
**Average mental health and stressor load across T0–T3**						
Inverted GSI of the BSI-18, *M* (*SD*)	66.95 (3.00)	61.43 (5.28)	54.48 (6.65)	44.29 (6.22)	$\chi_{\left(3\right)}^{2}$ = 74.84	**<0.001**
WHO-5, *M* (*SD*)	69.39 (8.14)	51.19 (9.37)	43.81 (9.16)	31.83 (9.78)	*F*_(3, 129)_ = 71.51	**<0.001**
Frequency of microstressor encounters, *M* (*SD*)	48.39 (21.32)	49.98 (22.09)	55.58 (21.43)	62.10 (24.51)	$\chi_{\left(3\right)}^{2}$ = 4.42	0.219
Count of stressful life events, *M* (*SD*)	3.07 (1.47)	2.86 (1.45)	3.07 (1.26)	4.44 (1.96)	$\chi_{\left(3\right)}^{2}$ = 6.81	0.078
**Resilience factors and perceived stress during the pandemic (T4)**						
Optimism, *M* (*SD*)	9.09 (1.87)	7.14 (2.39)	6.06 (2.96)	5.36 (2.06)	*F*_(3, 113)_ = 10.61	**<0.001**
Self-care, *M* (*SD*)	4.10 (0.70)	3.66 (0.74)	3.51 (1.10)	2.83 (0.56)	$\chi_{\left(3\right)}^{2}$ = 20.17	**<0.001**
Perceived emotional support, *M* (*SD*)	14.75 (1.44)	14.21 (2.12)	13.11 (3.41)	12.45 (2.38)	$\chi_{\left(3\right)}^{2}$ = 8.68	0.034
Generalized self-efficacy, *M* (*SD*)	31.59 (3.16)	28.46 (3.46)	27.17 (6.02)	24.64 (2.94)	$\chi_{\left(3\right)}^{2}$ = 28.06	**<0.001**
Perceived stress, *M* (*SD*)	19.66 (2.78)	21.48 (3.51)	21.00 (3.53)	23.91 (2.17)	*F*_(3, 113)_ = 5.19	**0.002**

*Inverted GSI of the BSI-18, inverted Global Severity Index of the Brief Symptom Inventory-18 (higher scores indicate better mental health); WHO-5, World Health Organization Well-Being Index. Where the requirements for analysis of variance (ANOVA) were not met, the Kruskal-Wallis rank sum test was used instead. For data assessed during the pandemic, 117 participants provided complete data and were included in the analyses. To account for multiple tests, we set the significance at a Bonferroni adjusted alpha level of 0.0125 for analyses of baseline and T0-T3 data and 0.01 for analyses of pandemic data. All significant p-values were bolded*.

### Predictors of Resilient Functioning Trajectory Class

We investigated the resilience factors (optimism, self-care, social support, and generalized self-efficacy) assessed at T0 as predictors of most likely latent class membership in a multinomial logistic regression analysis. Data screening revealed no apparent problems in terms of extreme outliers or multicollinearity among predictors. Overall, the model demonstrated satisfactory fit [$\chi_{\left(21\right)}^{2}$ = 68.05, *p* <0.001; McFadden *R*^2^ = 0.21], correctly classifying 60% of the cases. However, prediction accuracy was not very good for smaller classes, likely reflecting unbalanced class sizes and ultimately sample size constraints. Whereas, 82% of participants assigned to the medium trajectory class and 53% of participants assigned to the high trajectory class were classified correctly, the rate of correct classification for the low and medium-to-low trajectory classes was only 33 and 24%, respectively. We set the low trajectory class as the reference category, comparing each of the other classes to this group. Only self-care emerged as a significant predictor of latent class membership. Compared to the low trajectory class, participants in the high and medium-to-low classes engaged in more self-care. Detailed results for all predictors included in the model are reported in Table 3.

**Table 3:** Multinomial logistic regression results for predicting resilient functioning trajectory class.

	**High vs. low**			**Medium vs. low**			**Medium-to-low vs. low**		
**Predictor**	***B* (*SE*)**	***OR* (95% *CI*)**	***p***	***B* (*SE*)**	***OR* (95% *CI*)**	***p***	***B* (*SE*)**	**OR (95% CI)**	***p***
Optimism	0.29 (0.21)	1.34 (0.89-2.02)	0.157	0.14 (0.18)	1.15 (0.81-1.63)	0.450	0.10 (0.20)	1.10 (0.75-1.63)	0.621
Self-care	2.69 (0.90)	14.80 (2.56-85.68)	**0.003**	1.19 (0.74)	3.28 (0.77-13.93)	0.107	2.49 (0.90)	12.06 (2.07-70.32)	**0.006**
Social support	0.58 (0.86)	1.78 (0.33-9.63)	0.504	0.32 (0.73)	1.37 (0.33-5.71)	0.661	−0.10 (0.80)	0.91 (0.19-4.36)	0.903
Self-efficacy	0.20 (0.15)	1.22 (0.90-1.64)	0.192	0.20 (0.14)	1.22 (0.93-1.61)	0.157	0.04 (0.15)	1.04 (0.77-1.40)	0.817
Age	−0.08 (0.24)	0.93 (0.58-1.48)	0.750	−0.07 (0.22)	0.93 (0.61-1.42)	0.745	0.09 (0.23)	1.09 (0.70-1.69)	0.704
Gender	−1.79 (1.22)	0.17 (0.02-1.81)	0.141	−1.09 (1.15)	0.34 (0.04-3.24)	0.347	−0.99 (1.25)	0.37 (0.03-4.31)	0.429
Cohort	−1.56 (1.05)	0.21 (0.03-1.63)	0.135	−1.62 (0.96)	0.20 (0.03-1.29)	0.090	0.17 (1.21)	1.19 (0.11-12.63)	0.885

*B, parameter estimate; SE, standard error; OR, odds ratio; CI, confidence interval. All significant p-values (p <0.05) were bolded*.

### Stress and Mental Health During the Lockdown

We analyzed data across the whole sample to characterize the impact of the COVID-19 pandemic, irrespective of resilient functioning trajectory class. Most of the participants agreed with government-mandated restrictions (*M* = 4.22, *SD* = 0.81) and reported that they followed recommendations to contain the spread of the virus (*M* = 4.50, *SD* = 0.57). Of 29 pandemic-specific stressors, participants experienced on average 10.4 (*SD* = 3.20; intensity: *M* = 3.23, *SD* = 0.65) with the most frequent being media reports (100%), loss of opportunity for recreational activities (97%), and loss of social contacts (90%). PSS scores indicated moderate levels of perceived stress overall (*M* = 21.14, *SD* = 3.39). Comparisons of data assessed at T3 and T4 showed no significant change in the frequency of microstressors [*t*_(116)_ = 1.38, *p* = 0.170] or the count of stressful life events [*t*_(116)_ = −1.08, *p* = 0.283]. Note that at T4 participants reported on stressful life events in the past 3 months (i.e., including the weeks before the pandemic). The comparison of inverted GSI scores of the BSI-18, assessed during and before the pandemic, revealed a significant decrease in symptoms during the lockdown [*t*_(116)_ = −2.71, *p* = 0.008]. Correspondingly, WHO-5 scores showed a significant increase [*t*_(116)_ = −2.17, *p* = 0.032], reflecting improved well-being during the lockdown.

### Predicting Resilient Functioning During the Lockdown

Using multiple regression analysis, we examined resilient functioning trajectory class, resilience factors, and perceived stress as predictors of resilient functioning during the pandemic. Importantly, we focused on indices of optimism, self-care, perceived emotional support, and generalized self-efficacy re-assessed during the lockdown (see Table 2 for descriptive statistics by trajectory class). The data were checked for extreme outliers and parametric assumptions, with no apparent problems. There was also no evidence of multicollinearity among predictors (generalized variance inflation factors <3; see Supplementary Table 2 for zero-order correlations among predictors). All continuous predictors were mean centered and the reference level for resilient functioning trajectory class was set to the low trajectory class. The overall model was significant [*F*_(8, 108)_ = 20.20, *p* <0.001] and the adjusted *R*^2^ indicated that 57% of the variation in resilient functioning was accounted for. We conducted a separate regression that included age, gender, and cohort as covariates, but a model comparison indicated no significant improvement [*F*_(3, 105)_ = 1.07, *p* = 0.363]. We therefore retained the more parsimonious model with six predictors. In this model, trajectory class, self*-*care, and perceived stress emerged as significant predictors (see Table 4 for all results). Since the assumptions for ANOVA were not met, we conducted a Kruskal-Wallis rank sum test and Dunn's test with Holm-Bonferroni correction to follow up on class-dependent differences in resilient functioning. Results revealed significant differences [$\chi_{\left(3\right)}^{2}$ = 53.37, *p* <0.001, η^2^_*H*_ = 0.44] with participants in the high trajectory class showing significantly higher resilient functioning than participants in all other classes (all *p* <0.001) and participants in the low trajectory class showing lower resilient functioning than participants in all other classes (low vs. medium: *p* = 0.008; low vs. medium-to-low: *p* = 0.075). The contrast of medium vs. medium-to-low (*p* = 0.456) was not significant. More self-care and lower perceived stress was predictive of higher resilient functioning scores. Descriptive statistics (Table 2) indicated that participants in the high trajectory class took greater care of themselves during the pandemic compared to participants in the other classes, especially those in the low trajectory class. Hence, including an interaction term of class x self-care in the regression model yielded significant improvement in model fit [*F*_(3, 105)_ = 3.93, *p* = 0.011] and the coefficient for medium-to-low class × self-care was significant (*p* = 0.031). This improved model explained 60% variation in resilient functioning during the lockdown (see Table 4 for results of both models).

**Table 4:** Multiple regression results showing predictors of resilient functioning during the lockdown.

**Predictor**	***B* (95% *CI*)**	***SE***	**β**	***t***	***p***
**Model without interaction**					
Resilient functioning trajectory					
High	1.62 (0.98–2.25)	0.32	0.63	5.07	**<0.001**
Medium	0.83 (0.29–1.37)	0.27	0.36	3.06	**0.003**
Medium-to-low	0.68 (0.07–1.28)	0.30	0.21	2.22	**0.028**
Optimism	0.05 (−0.03–0.13)	0.04	0.11	1.17	0.244
Self-care	0.35 (0.12–0.58)	0.11	0.25	3.06	**0.003**
Perceived emotional support	0.01 (−0.06–0.08)	0.04	0.02	0.34	0.735
Generalized self-efficacy	0.03 (−0.02 to −0.07)	0.02	0.11	1.24	0.217
Perceived stress	−0.05 (−0.09 to −0.00)	0.02	−0.14	−2.11	**0.037**
*R*^2^ (adjusted)	0.60 (0.57)				
*F*	20.20				
*P*	**<0.001**				
**Model with interaction**					
Resilient functioning trajectory					
High	2.35 (1.38–3.33)	0.49	0.91	4.78	**<0.001**
Medium	1.38 (0.48–2.29)	0.46	0.60	3.04	**0.003**
Medium-to-low	1.27 (0.33–2.20)	0.47	0.40	2.68	**0.009**
Optimism	0.02 (−0.06–0.10)	0.04	0.04	0.38	0.704
Self-care	−0.18 (−1.058–0.69)	0.44	−0.13	−0.41	0.681
Perceived emotional support	0.00 (−0.07–0.07)	0.03	0.00	0.02	0.984
Generalized self-efficacy	0.02 (−0.02–0.07)	0.02	0.09	1.10	0.274
Perceived stress	−0.05 (−0.09–0.00)	0.02	−0.14	−2.11	**0.037**
Interaction: resilient functioning trajectory × self-care					
High × self-care	0.28 (−0.69–1.25)	0.49	0.10	0.58	0.567
Medium × self-care	0.52 (−0.40–1.45)	0.47	0.23	1.12	0.266
Medium-to-low × self-care	1.10 (0.10–2.03)	0.49	0.38	2.19	**0.031**
*R*^2^ (adjusted)	0.64 (0.60)				
*F*	16.96				
*P*	**<0.001**				

*Dependent variable: resilient functioning during the pandemic. The reference category for resilient functioning trajectories was the low trajectory class. All significant p-values (p < 0.05) were bolded*.

## Discussion

In this study, we explored students' resilient functioning over a 9-month period before the outbreak of the COVID-19 pandemic and investigated links with baseline assessments of established resilience factors and with resilience during the first pandemic-related lockdown in Germany. Four distinct trajectories of pre-pandemic resilient functioning were identified: high, low, medium, and medium-to-low (i.e., progressive decline). Most participants' trajectories could be described as rather stable at expected levels of resilience. Given that we operationalized resilient functioning as the residual resulting from the regression of mental health on stressor load, it was expected that most participants would fall close to the regression line. However, the second-largest class showed higher than expected levels of resilient functioning and only a small proportion of the sample demonstrated markedly lower resilience. In line with this, studies tracking the course of psychological outcomes in the wake of a traumatic event (58–60), generally find that most participants maintained good levels of mental health. Moreover, because we focused on trajectories of resilience during everyday life, we observed relatively low counts of stressful life events that could affect students' mental health. In fact, trajectories seemed to be best distinguished by intercept rather than slope, suggesting little perturbation by stress. Although university students have been reported to show increased prevalence rates of anxiety and depression (9–11), we had initially screened potential participants for eligibility for the intervention study, thus our sample represents rather healthy students. They may not have faced very severe stressors or already have adaptive strategies at hand for coping with stress. Indeed, our analyses showed that participants with consistently high levels of resilient functioning scored highest on optimism, self-care, social-support, and perceived self-efficacy while participants with markedly lower levels of resilient functioning scored lowest. This confirms our expectation that higher expressions in these established resilience-promoting factors should go along with better mental health despite stress. However, in a multinomial logistic regression, only self-care emerged as a significant predictor of resilient functioning trajectory class. Self-care generally describes health-promoting behaviors, such as adequate sleep, healthy eating, exercise, and relaxation (61, 62). Previous research in different student populations has linked greater engagement in self-care to lower levels of stress and greater well-being (63–66). Self-care has also been reported to weaken the association between stress and quality of life (67). Our findings are in line with this and expand upon existing research in students by focusing specifically on resilience.

To investigate the effects of the pandemic as a global stressor, we first sought to assess all students' perceived stress as well as potential changes in their mental health and stressor load compared to the last assessment prior to the lockdown (independent of resilient functioning trajectory). All participants reported having experienced some pandemic-specific stressors, such as alarming reports by the media, but levels of perceived stress were moderate overall. We did not observe any significant increase in stressor load, nor any decrease in mental health. On the contrary, participants reported less symptoms of mental health problems and increased well-being during the pandemic. This was unexpected but ties in with a previous report on changes from before the pandemic to the first lockdown in a sample of the general population in Germany (68). Kohls et al. (69) provided a very comprehensive picture of over 3,000 university students assessed during this lockdown. According to their reports, more than half of the sample did not feel personally affected by the pandemic at that time and a majority perceived not only negative, but also positive aspects. In line with this, Ahrens et al. (68) discuss the concept of psychosocial gains from adversity (70), surmising that the pandemic, as a collectively experienced adverse event, may have strengthened social bonds.

Critically, the picture is very different when considering other populations. In March 2020, the WHO issued advice on how best to deal with the pandemic, paying particular attention to groups such as healthcare staff, carers of children, or older adults (71). Many studies have reported alarming rates of stress and mental health problems among healthcare workers during the first wave of the pandemic (72–74). It is crucial to note, however, that the first wave of the pandemic is unlikely to be representative of how students fared during subsequent waves. To date, there is a lack of research on stress and well-being during the later stages of the pandemic, but large-scale longitudinal projects are ongoing (75). In a preprint, Shevlin et al. (76) observed that most people consistently showed low levels of anxiety and depression and trajectories appeared to stabilize over the first year of the COVID-19 pandemic. Similarly, Prati and Mancini (77) noted that lockdowns did not show uniformly detrimental effects and most people appeared resilient. However, based on a systematic review of the prevalence of post-traumatic stress disorder in the wake of infectious disease pandemics in the 21st century, Yuan et al. (78) cautioned that more studies with longer follow-up times were needed to fully characterize the impact of this pandemic.

We found previous levels of resilient functioning to be predictive of students' resilience during the first lockdown. Specifically, those who had exhibited consistently high resilience and engaged in more self-care behaviors during the pandemic were characterized by high resilient functioning. However, given that students in our sample did not appear to be negatively affected by the lockdown and considering that self-care predicted higher resilience at baseline (i.e., several months before the outbreak of the pandemic), we cannot be sure that self-care presents a critical factor in dealing with pandemic-related stressors. Rather, it seems that self-care is generally important for mental health in the face of everyday life stress. It may be, however, that self-care played a more important role than other resilience factors during the first lockdown. After all, pandemic events were largely uncontrollable and associated with a loss of social contacts, thus, active coping driven by high perceived self-efficacy and the maintenance of social networks was complicated. This may have brought the self and, in turn, self-care behaviors to the fore. In general, intervention studies aiming to boost self-care behaviors, could show significant reductions in stress and depressed mood (79, 80). In cross-sectional studies comparing different coping strategies and protective factors during the initial stages of the pandemic, keeping regular routines, going outside, and limiting screen time emerged as particularly effective health-promoting behaviors (13, 81). Ornell et al. (82) also list self-care behaviors among mental health recommendations during pandemics. Given the severe strain healthcare workers in particular are under, psychosocial support programmes have been called for (83) and some hospitals have acted quickly to implement appropriate measures (84). Notably, many have advocated for organizations to promote self-care as an effective strategy to reduce stress and prevent mental health problems (85–87). Blake et al. (88) developed a digital learning package to promote well-being in healthcare workers during the pandemic, involving healthcare staff in the design process. A core component of the resulting package forms strategies for better self-care, underscoring the importance of self-care for mental health. Future studies should therefore determine the critical contribution of self-care to resilient functioning in students, especially during the later stages of the pandemic.

Some limitations must be considered when interpreting the findings of this study. First, our sample was rather small and the observed effects may therefore not be very robust. Given the unexpected and unprecedented outbreak of the COVID-19 pandemic, most studies that investigated its effects are cross-sectional in nature. Since we had data on students' stress and mental health available from before the pandemic, we focused on following up with these participants. While this restricted our sample size, our findings can shed further light on how characteristics assessed prior to the pandemic link to students' response to the first lockdown. Second, we are lacking potentially very interesting follow-up data from subsequent waves of the pandemic. Third, our sample is selective since we only assessed students from one university in Germany. Research has uncovered striking differences in the impact of the pandemic by country (89, 90), and initial regulations in Germany were county-specific (91), complicating comparisons even between different regions within the country.

Although findings should be interpreted with caution, they represent a rare examination of established resilience factors in relation to resilience over an extended time period and highlight the relevance of self-care in coping with real-life stressors such as the pandemic.

## Data Availability Statement

Data and code (including Mplus files) are available on the Open Science Framework: https://osf.io/embcr/.

## Ethics Statement

The studies involving human participants were reviewed and approved by the Ethics Committee of the Institute of Psychology, Johannes Gutenberg-University, Mainz, Germany. The participants provided their written informed consent to participate in this study.

## Author Contributions

LM and MW: conceptualization. LM: methodology, software, formal analysis, writing-original draft preparation, and visualization. ES, SS, EE, and AB: investigation. LM and ES: data curation. LM, ES, and MW: writing-review and editing. MW: supervision and funding acquisition. ES, SS, EE, AB, and MW: project administration. All authors contributed to the article and approved the submitted version.

## Funding

This research was funded by the State of Rhineland-Palatinate and the Leibniz-Institute for Resilience Research.

## Conflict of Interest

The authors declare that the research was conducted in the absence of any commercial or financial relationships that could be construed as a potential conflict of interest.

## Publisher's Note

All claims expressed in this article are solely those of the authors and do not necessarily represent those of their affiliated organizations, or those of the publisher, the editors and the reviewers. Any product that may be evaluated in this article, or claim that may be made by its manufacturer, is not guaranteed or endorsed by the publisher.
