# Supplementary material for: Look After Yourself: Students Consistently Showing High Resilience Engaged in More Self-Care and Proved More Resilient During the COVID-19 Pandemic
Source: Front Psychiatry. 2021 Dec 21;12:784381. doi: 10.3389/fpsyt.2021.784381 (PMC8725157; doi:10.3389/fpsyt.2021.784381)
Supplement: Supplementary file 1 [file Data_Sheet_1.docx]

Supplementary Material

Supplementary Table 1. Linear regressions of mental health on stressor load at T0-T4

| **Time Point** | ***B*** | ***SE*** | ***t*** | ***p*** |
| --- | --- | --- | --- | --- |
| T0 | -0.40 | 0.09 | -4.25 | < .001 |
| T1 | -0.44 | 0.08 | -5.30 | < .001 |
| T2 | -0.38 | 0.08 | -4.54 | < .001 |
| T3 | -0.45 | 0.08 | -5.56 | < .001 |
| T4 | -0.37 | 0.09 | -4.12 | < .001 |

*Note.* Following separate principal component analysis of variables reflecting mental health (inverted Global Severity Index of the Brief Symptom Inventory and World Health Organisation Well-Being Index) and stressor load (frequency of microstressor encounters and count of stressful life events), the first mental health component was regressed on the first stressor load component. *N* = 133 for T0-T3 and *N* = 117 for T4.


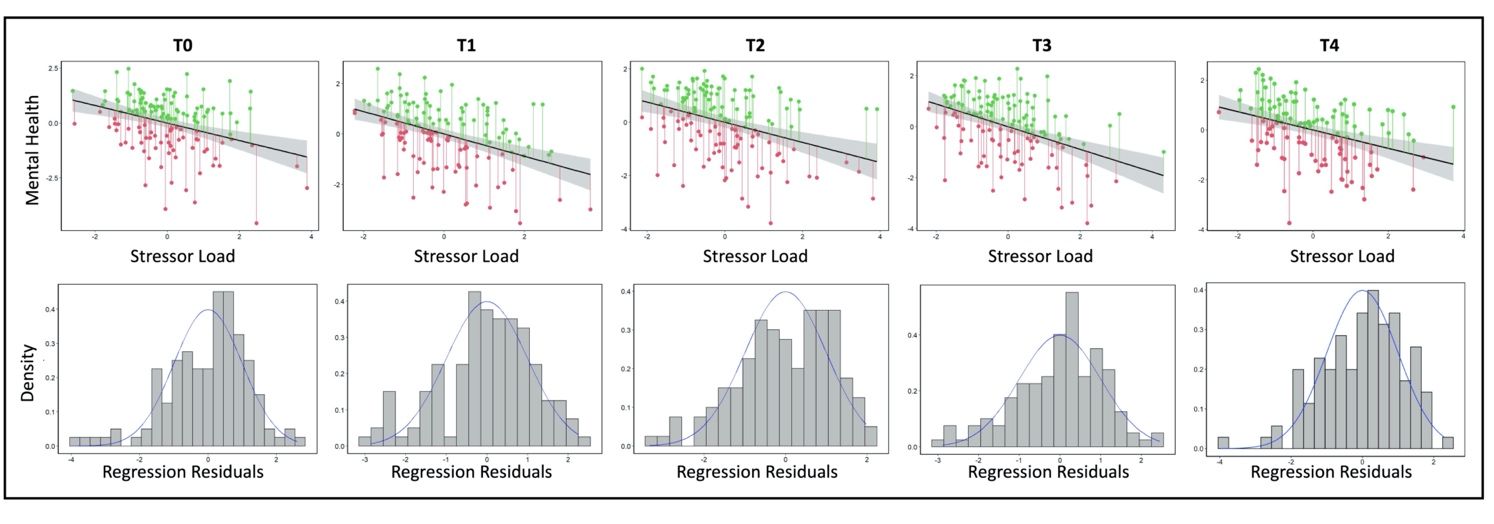


**Supplementary Figure 1.** Linear regression of mental health on stressor load depicted in the top row for T0-T4. Residuals marked in green lie above the regression line and represent better than expected mental health given stressor load, i.e., higher resilient functioning. Residuals marked in red lie below the regression line and are therefore taken to reflect lower resilient functioning. Histograms of the residuals for T0-T4 are displayed in the bottom row and indicate approx. normally distributed scores in each case.


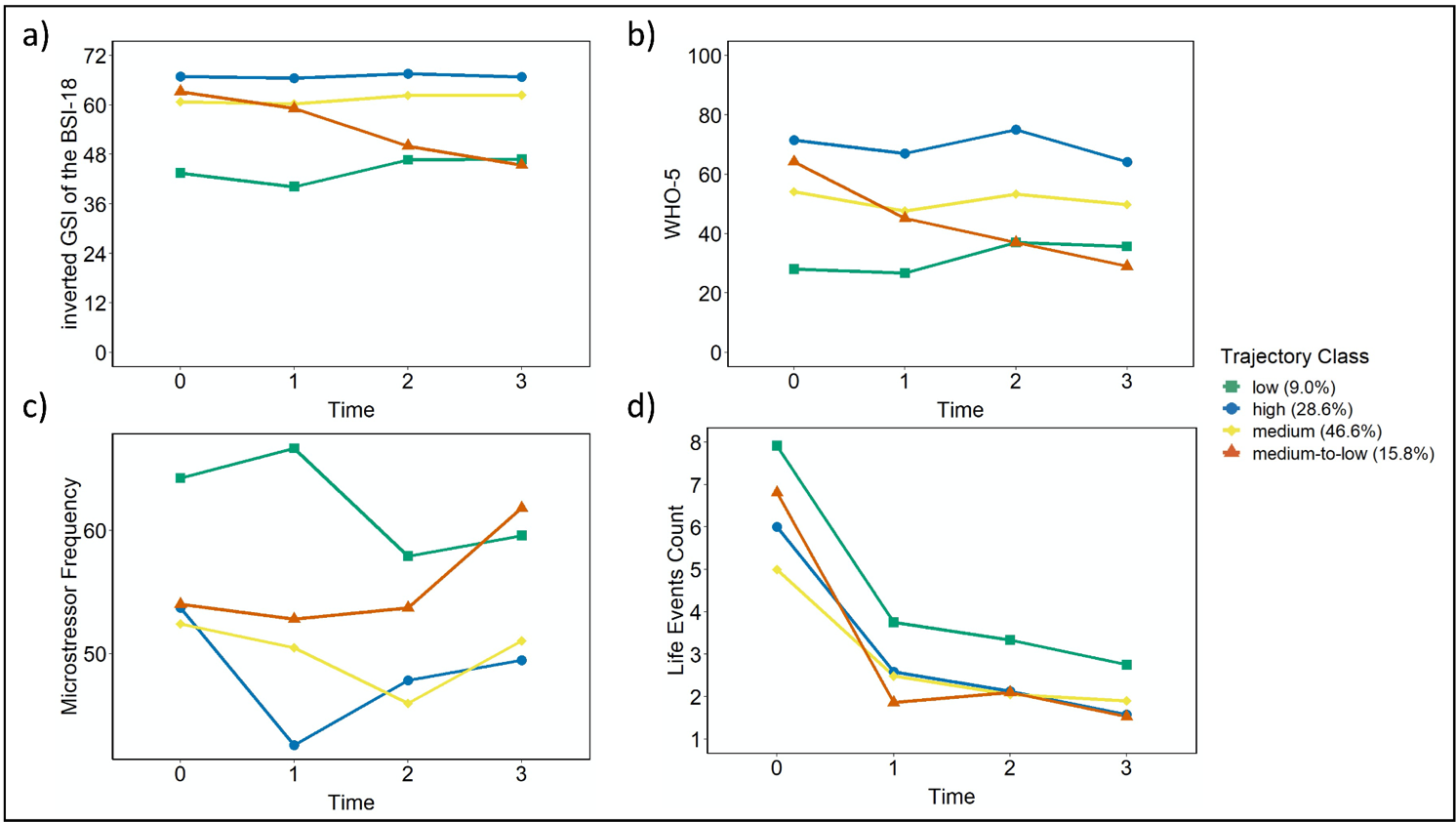


**Supplementary Figure 2.** Mental health and stressor load of the four latent classes from T0-T3. a) Inverted GSI of the BSI-18 = inverted Global Severity Index of the Brief Symptom Inventory-18 (higher scores indicate better mental health), b) WHO-5 = World Health Organization Well-Being Index, c) Frequency of microstressor encounters, d) count of stressful life events (T0 includes any events experienced up to this time point, whereas T1-T3 refer to events experienced within the past three months).


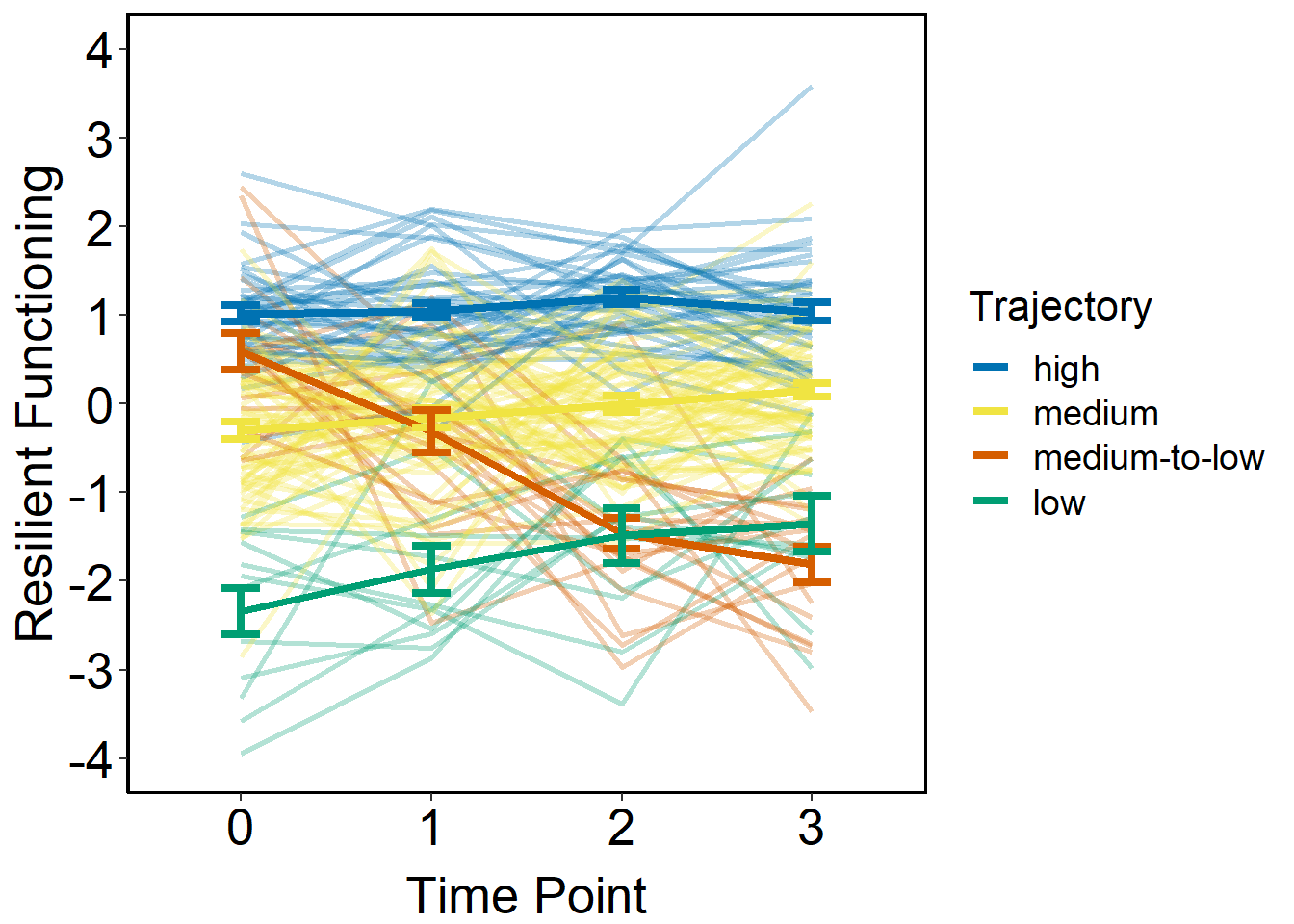


**Supplementary Figure 3.** Individual resilient functioning trajectories and class means from T0-T3.

Supplementary Table 2. Zero-order correlations of resilient functioning predictors at T4

|  | Optimism | Self-care | Perceived emotional support | Generalized self-efficacy | Perceived stress |
| --- | --- | --- | --- | --- | --- |
| Optimism | 1 |  |  |  |  |
| Self-care | **0.59***** | 1 |  |  |  |
| Perceived emotional support | **0.32***** | **0.44***** | 1 |  |  |
| Generalized self-efficacy | **0.66***** | **0.44***** | **0.21*** | 1 |  |
| Perceived stress | -0.15 | **-0.25**** | 0.04 | **-0.29**** | 1 |

*Note. N* = 117. * *p* < 0.05, ** *p* < 0.01, *** *p* < 0.001
